# Technoscientia est Potentia? Contemplative, interventionist, constructionist and creationist idea(l)s in (techno)science

**DOI:** 10.1007/s10202-011-0101-2

**Published:** 2011-11-30

**Authors:** Karen Kastenhofer, Jan C. Schmidt

**Affiliations:** 1 Institute of Technology Assessment, Austrian Academy of Sciences, Strohgasse 45/5, 1030 Wien, Austria; 2 Unit of Social, Culture and Technology Studies, Darmstadt University of Applied Sciences, Haardtring 100, 64295 Darmstadt, Germany

## Abstract

Within the realm of nano-, bio-, info- and cogno- (or NBIC) technosciences, the ‘power to change the world’ is often invoked. One could dismiss such formulations as ‘purely rhetorical’, interpret them as rhetorical *and* self-fulfilling or view them as an adequate depiction of one of the fundamental characteristics of technoscience. In the latter case, a very specific nexus between science and technology, or, the epistemic and the constructionist realm is envisioned. The following paper focuses on this nexus drawing on theoretical conceptions as well as empirical material. It presents an overview of different technoscientific ways to ‘change the world’—via contemplation and representation, intervention and control, engineering, construction and creation. It further argues that the hybrid character of technoscience makes it difficult (if not impossible) to separate knowledge production from real world interventions and challenges current science and technology policy approaches in fundamental ways.

## Résumé

Dans le monde des nano-, bio-, info- et cogno- (ou NBIC) technosciences, le «pouvoir de changer le monde» est souvent invoqué. Ces formulations pourraient être mises de côté comme «purement rhétoriques», ou bien interprétées comme étant rhétoriques et auto-réalisatrices, ou bien vues comme une description adéquate d’une des caractéristiques fondamentales de la technoscience. Dans le dernier cas, une connexion bien spécifique est imaginée entre la science et la technologie, ou bien le monde épistémique et obstructionniste. Cet article se concentre sur cette connexion, basé sur des conceptions théoriques ainsi que du matériel empirique. Il présente une vue d’ensemble des différentes manières de «changer le monde»— via la contemplation et la représentation, l’intervention et le contrôle, l’ingénierie, la construction et la création. De plus, l’étude soutient que le caractère hybride de la technoscience rend difficile (voire impossible) de séparer la production de la connaissance des interventions du monde réel et remet en question d’une manière fondamentale les approches actuelles de la politique de la science et de la technologie.

## Abstract (de)

In Darstellungen der neuen Nano-, Bio-, Info- und Kogno-Technowissenschaften wird häufig deren „weltverändernde Macht“ beschworen. Man kann solche Formulierungen nun als „rein rhetorische Figur“ abtun, sie als rhetorisch und selbstbestätigend werten oder auch als treffende Charakterisierung von Technowissenschaft verstehen. In letzterem Fall wird eine sehr spezifische Verknüpfung von Wissenschaft und Technologie, dem epistemischen und dem konstruierenden Bereich thematisiert. Der vorliegende Artikel widmet sich dieser Verknüpfung von theoretischer und empirischer Seite. Er stellt unterschiedliche technowissenschaftliche Ansätze dar „die Welt zu verändern“—durch Kontemplation und Repräsentation, Intervention und Kontrolle, Konstruktion und (Neu-)Schöpfung. Der hybride Charakter von Technowissenschaften—so die weitere Folgerung—erschwert die praktische Trennung von Wissensproduktion und gesellschaftlicher Intervention und stellt somit Fundamente herkömmlicher Wissenschafts- und Technologiepolitik in Frage.

## Introduction

Maybe there are two distinct mythical origins of the idea of ‘reality’. One is the reality of representation, the other the idea of what affects us and what we can affect. (…) We shall count as real what we can use to intervene in the world to affect something else, or what the world can use to affect us. Reality as intervention does not even begin to mesh with reality as representation until modern science. Natural science since the seventeenth century has been the adventure of the interlocking of representing and intervening. It is time that philosophy caught up to three centuries of our own past. (Hacking 1983: 146)

When Ian Hacking formulated his now famous treatise on “Representing and Intervening”, he referred to an idea(l) of science that is based upon the ambition to represent the natural world via the formulation of theoretical concepts and natural laws on the one hand and supplemented it with an idea(l) that puts intervention centre stage in its experimental research practices on the other hand. In Hacking’s text, representing and intervening are depicted as two scientific modes of addressing the natural world that have been ‘interlocked’ since the emergence of modern science. He further points out that scientists have been aware of the resulting hybrid character of their actions for a long time, while philosophers of science neglected the interventionist aspect of science, focusing solely on its representational character. His plea to acknowledge both aspects of the relation between science and reality when discussing and analysing modern science—its representational as well as interventionist character—is thereby evocative of the emphasis on the hybridity of current science captured by the term ‘technoscience’.^1^ The label ‘technoscience’ explicitly refers to a technological/interventionist as well as a scientific/representational stance; the “interlocking” of both stances and its scientific as well as societal consequences are central themes within the technoscience literature (classical examples include Hottois 1984; Latour 1987; Haraway 1997).

Nevertheless, in Hacking’s treatise as well as in the technoscience literature, both stances or (techno)science idea(l)s^2^ are still mentioned separately—albeit all interlocking, hybridity and amalgamation hypotheses. The “two distinct mythical origins”, the two ideal views of what science is or ought to be in general seem to be more alive then ever.^3^ Almost every statement referring to an aspect of science, be it science as an institution, science as a body of knowledge or science as a research practice, includes explicit or implicit references to one or the other ideal of (techno)science. The two idea(l)s of (techno)science delineate specific relations between science, nature and society, building upon specific ideas not only about science, but also about nature and society.^4^ (Techno)science is characterised either as autonomous from, reactive to or embedded in other societal spheres; (techno)scientific knowledge either points towards a theoretical understanding of nature, general visions of the world we live in or technical know-how to change our living conditions; research practice is described either as “reading in the Book of Nature”, “experimenting with Nature” or “constructing artefacts”. But what is the relation between the “two distinct mythical origins” and current (techno)science, between the (techno)scientists’ (or a society’s) idea about science, (techno)scientific practice and its societal impact?

It is the main thesis of this paper that such formulations point towards ideal pictures that, by amalgamating how the world looks like, how it is performed and how it should look like, exert an influence on many different levels; they pre-configure discourses, practices and socio-epistemic settings and are themselves realised and reaffirmed by them; they shape not only how we *speak about* science in research funding applications and programmes or in science studies, but also how science is *done* in research laboratories and elsewhere, how future scientists are educated and how scientists, science and its products come to matter in social contexts. To address this main thesis, this paper focuses on three central questions: What exactly are the contemporary ideal pictures of (techno)scientific practice (like, for instance, representation of and intervention in the natural world)? How can the influence of (techno)science idea(l)s on doing (techno)science be conceptualised (beyond a mere juxtaposition of *talking* versus *doing* science)? What is their relevance in the socio-political context? Based on the discussion of these questions, possible differences between science and technoscience are addressed that have that far been neglected by talking about (techno)science in an undifferentiated manner.

Following this rough framework, we shortly introduce two approaches that try to address the inter-relation between ideal pictures of (techno)science and doing (techno)science in chapter 2. In chapter 3, we take a closer look at the (techno)science idea(l)s put forward in the research programmatics of Francis Bacon, Vannevar Bush and the new nano-, bio-, info- and cogno-technoscience (NBIC) initiatives. In chapter 4, we reconstruct (techno)science idea(l)s present in doing (techno)science. We argue that there are more idea(l)s of (techno)science out there than the idea(l) of science as representing on the one hand and the idea(l) of science as intervening on the other hand; we also claim that the interpretive idealist stances and the material-epistemic practices of (techno)science do not inhibit totally separate worlds (more or less in line with Hacking’s position). Some material-epistemic actions are closer to one stance than others (i.e. they are more easily made sense of by this stance than by another stance due to specific general perceptions prevalent in a given culture) and this holds true even more so for combinations of material-epistemic actions that emerge and are stabilised based upon specific interpretations and idea(l)s. To illustrate our position, we will draw on various socio-historical contexts, contrasting earlier developments with current situations, as well as on various epistemic cultures, contrasting different research practices and different actors’ accounts of research. In our conclusion in chapter 5, we will reconsider the relation between ideal types of (techno)science, (techno)scientific research practice and the societal significance of (techno)science as a means to address the relation between science, technology and technoscience.

## Capturing the relation of science discourse, science idea(l)s and scientific practice

Within current technoscience analyses—sociological, philosophical or political studies of nanotechnology, synthetic biology, neuro-technoscience or converging technologies—the analysis of *discourses* about these technosciences in public, often hybrid arenas (such as technoscience funding proposals and programmes, governance discourse, regulatory controversies) take a more and more prominent role. Talk *about* science seems to have become more interesting for critical science studies than the technoscientific practices and potential societal impacts of the technosciences themselves. Other scholars follow a more traditional approach and analyse technoscience from an epistemological, micro-sociological or technology assessment approach. From their perspective, the new technosciences do not differ radically from any other sciences or they consist of mere hypes and buzzwords, in short, they represent “empty signifiers” (cp. Wullweber 2008). Very seldom the challenge to analyse the relation between (techno)science discourses, prevalent conceptions of (techno)science and (techno)scientific practice and products is taken up. Hacking’s (1983) reconstruction of the two idea(l)s of science, science as representing or intervening, could serve as a good starting point to draw connections between these often actively differentiated dimensions. Although analyses of science-associated ideals (immanent in a scientific habitus or thought style, practical norms or institutional decisions) and their effects on science (individual interpretive approaches, the collaborative fabrication of knowledge as well as the collective organisation of science) have been undertaken throughout the histories of epistemology, social studies of science and laboratory ethnography, further theoretical elaborations that would help to conceptualise the relation of discourse and practice for the current technosciences are still lacking.

Lyotard (1982/1993) was one of the first authors to use the term technoscience in his analyses. When doing so, he also introduced the notion of Ideas,^5^ which—in his view—govern different domains. He identifies four domains within the most developed societies, namely the scientific, the technical, the economic and that of the state.

Each of these domains, which is closely interwoven with the others, is distinguished only insofar as each domain is governed by a different Idea [or: ideal]; the scientific is governed by the Idea of the best knowledge, the technical by that of optimum performance (the best input/output ratio), the economic by the Idea of the highest wealth, the state by the Idea of the best being-together. Each of these Ideas is an absolute towards which one has to work. The Idea has a regulatory function for the discourses and actions occurring in each of the aforementioned domains.

He goes on to note that the agents’

phrases and their acts ask to be evaluated according to the criterion that corresponds to the regulatory Idea of their proper domain. In the present epoch, science and technology combine to form contemporary technoscience. In technoscience, technology plays the role of furnishing the proof of scientific arguments. It allows one to say of a scientific utterance that claims to be true, ‘here is a case of it.’ The result of this is a profound transformation in the nature of knowledge. Truth is subjected to more and more sophisticated means of ‘falsifying’ scientific utterances. (ibid: 14, 15)

“The four Ideas”, he adds in his conclusion, “are not descriptions of realities, but regulatory Ideas (containing a prescription).” (ibid: 18) The concept of regulatory Ideas refers to Kant’s (1989/1787) “*Regulatory Ideas of Reason*” such as ‘Time’, ‘Space’ and ‘Causation’. These are conceived as cognition related entities that guide how we address the (natural) world but are not part of the (natural) world. Kant’s regulatory Ideas belong to the realm of reason (‘Vernunft’), not that of experience (‘Wahrnehmung’) or mind (‘Verstand’). This conception of regulatory Ideas thereby allows for the acknowledgement of both, a (material) reality and the specific conceptions someone holds, and tries to delineate the relation between both.^6^

Current ideas about the position and character of science within society can be said to exert a similar kind of influence as the one outlined by Lyotard. Furthermore, they are closely linked to other ideas—such as ideas about objectivity and about nature—that constitute, affirm, represent and influence them. Together, they constitute an a priori (as well as an a posteriori) of epistemic practice.^7^ Practice relevant ideas also delineate a gradient from a mostly normatively applied concept (what is to be conceived as sound science and under which conditions this ideal can be realised), via a normative-ontic idea (sound science should enact objectivity by specific means because of the nature of Nature) to an ontological conception (about the nature of research and the nature of Nature). Idea and ideal hence are closely linked. Moreover, it has to be noted that notions of science, objectivity and nature guide how scientists address the natural world, but they are not directly addressed by them, at least not under ‘normal’ circumstances or as part of ‘essential’ scientific education and practice [cp. Kastenhofer (2004a, b) for biology; that this situation varies between different epistemic cultures is addressed in Kastenhofer (2007)]. To implicitly acquire and individually hold a general and very abstract idea of sound science that can never be met completely seems to suffice to stabilise the collective undertaking of scientific research. In this way, science idea(l)s constitute part of the (cognitive) *hinterland* (Law 2004) of scientific research and its organisation. They are not directly addressed and cannot be proven or falsified, but function both as an a priori condition of reason and a product of epistemic practice (in the sense that they are enacted, stabilised and confirmed by successful action within research). These three aspects of regulatory Ideas—that they are never completely met in real situations, that they are somehow distanced from the directly observable and addressed reality and that they (co-)regulate our (cognitive or practical) actions—are also present in more recent studies of science and technology.

The notion of narratives has also been introduced to the realm of science studies in its own right. Lyotard (1979/2009) announces the collapse of Grand Narratives, including those concerning science (such as Enlightenment reductionism) and their substitution by a plurality of (Wittgensteinian) language games in post-modern societies. De-constructivist historians have since discussed narratives as epistemic tools (or cognitive *hinterland*) immanent in the meaningful (re-)construction of historical developments [cp. White’s (1990) analysis of different narratives within historical accounts] or natural phenomena [cp. Daston’s (1992) reconstruction of the emergence and impact of objectivity as a narrative]. Furthermore, the discursive level has been addressed by the frame-concept, which has been deployed in the study of technoscience in society in various ways—either as a kind of Kuhnian paradigm (in both definitions: as an epistemological predisposition *of* scientists or as an operational scheme *for* scientists^8^) or as a narrative element when talking *about* science [cp. Nisbet and Lewenstein’s (2002) analysis of the framing of biotechnology in media reports].

Nordmann (2010) recently deploys yet another formulation, namely that of an “organising myth”, in his analysis of technoscience. He puts the mythical character centre stage when stating that “the age of technoscience is just as mythical as was the one that preceded it”. Technoscience, along this outline, is not an object or a state, it is a vision; but it is not only a vision, it is an *organising* vision, a conviction that establishes alternative ideals and co-shapes agency (to an undetermined extent) via its influence on expectations and priorities: the

organising myth of technoscientific innovation orients the expectations and priorities of scientists and other social actors just as much or as little as did the powerful myth of science as a legacy of the Enlightenment (ibid: 6)

As a result, “the ‘technosciences’ do not even attempt to distinguish between theoretical representation of the world and technical intervention into the world”, whereas

the ‘pure’ sciences are pure precisely because they invest a lot of analytical effort into the conceptual and technical separation of these two activities. (…) The fact that the association of science, modernity and the Enlightenment is mythical does not make it less relevant for questions of self-image, cultural prestige and epistemic orientation of the many sciences and technosciences. (…) the abandonment of this myth is culturally significant for societies at large and for scientific self-understanding–especially when it comes to defending pure science and basic research, and especially when it comes to assessing the limits of knowledge or the relation between the regimes of truth and power. (ibid: 7, 8)

The effect of this new wording should not be underestimated: technoscience is not *only* a myth,^9^ it is an *organising* myth. Also, a myth is not *everything* there is^10^; myths are connected to other realms of reality in an organising way. The discursive and the practical meet eye to eye; they are (almost?) equally real and formative; they are each others’ *hinterlands*. When we conceive science not only as a system of absolute theoretical knowledge, but also as a practical, discursive and societal enterprise, ‘regulatory Ideas’, ‘ideal types’, ‘narratives’, ‘frames’, ‘paradigms’ and ‘organising myths’ within and about science not only enable and shape the perception and understanding of the world, but also how scientists interact with the world and how this interaction gains meaning. And the more the societal and scientific gaze shifts towards (the management of) a (a-historic) future (cp. Nordmann 2010), the more territory specific technoscientific narratives will gain as compared to epistemic and material practices and outcomes.

Conceptions of science as a representational undertaking and science as an enterprise of intervention can be understood as regulatory Ideas, ideal types, narrative frames or organising myths. The two accounts point towards different idea(l)s of science (based upon different depictions of what science is and/or should be about); they tell different stories about the relation between science and the physical world (representational or interventionist) which are sometimes connected to different societal roles and meanings of science (classical, humanistic science or powerful technoscience); and they can be treated as mutually exclusive in discourses about science (within philosophy of science, STS or science policy discourse). Perceived as idea(l)s, they are mutually exclusive in the sense that ideas are usually invested with absolute competence for the interpretation of the specific constellation they address (in the case of science ideals: the relation between science, the physical world and society) and that ideals can only guide actions and decisions when they are unequivocal. To explicitly hold two idea(l)s about the same constellation at the same time would hence be appraised as schizophrenic or at least ambiguous. Still, in everyday practice argumentative and material actions mirror plural orientations towards different idea(l)s within the same community or field of action all the time. These plural orientations are administered in various ways, for example by trying to reconcile different idea(l)s within a new comprehensive ideal, by differentiating different contexts of action and selectively attributing different idea(l)s to these different contexts and/or by explicitly thriving for a regime of plural idea(l)s. To link scientific knowledge production and technological power within a new paradigm has already been attempted by Bacon’s programme of combining “light bringing” and “fruit bearing”. Vannevar Bush in his 1945 research manifesto also makes a link between basic (representational) understanding and technical (interventionist) applications, but by instrumentally combining two different realms, not by epistem-ontological merging the two processes as if they were two sides of the same coin (or action). More recent manifestos, like the one put forward in Roco and Bainbridge’s (2002) report on “Converging Technologies for Improving Human Performance”, once again link scientific research with technological power and societal visions.

Moreover, actions taken in everyday practice may be inspired and shaped by specific idea(l)s and so is the way in which they are made sense of and their effects are taken up in further actions; but, following action theorists, they (and their effects) are not totally defined by these ideals or, more generally, by their situational interpretation. Hoyningen-Huene (1989: 51) points out this twofold character of actions when discerning (along Wright 1974) an internal and an external aspect of an operation or action whereby the internal aspect comprises the purpose of an action and a specific appraisal of a situation (herein lies the link to prevalent ideals) and the external aspect comprises the physical dimension of an action, i.e. the muscular activity and its known and intended impact. The thus circumscribed interaction of idea(l)s, actions and material practice becomes evident when looking at specific cases. Within everyday research practice, the two idea(l)s of representing and intervening (techno)science compete for the status of functioning as *the* ultimate ideal; but representing and intervening *actions* can still both be taken, made sense of and be referred to within one and the same epistemic system. This is possible because intervention can be understood as a means to a representational end and representation can aim towards methodical intervention. Hence, both actions can be linked to both idea(l)s in an instrumental way. The same research action can be interpreted either as interventionist when looking at its material practices, or as representational when focusing on its epistemic outcome. The latter focus is prominent in (idealist) accounts of basic or curiosity-driven research in the nineteenth and twentieth century; the former focus is prominent in accounts of the engineering sciences and also in accounts of experimental scientists who configure, set up and run experiments.^11^ Of late, the interventionist stance seems to have experienced a new career. Not only that representing and intervening have become acknowledged topics within the philosophy of science and that experimenting, instruments and epistemic objects gained increasing interest within science studies (cp. Latour 1987; Rheinberger 1997; Baird 2004)—that, in other words, Hacking’s plea has been complied with—also, public talk about science seems to have changed fundamentally. Rhetoric about emerging research fields’ “ability to understand *and* control”, “to flexibly manipulate bits, atoms, neurons and genes” and “to potentially create a new industrial revolution” abounds when the new NBIC technosciences are addressed. They are ascribed the “power to change the world as we know it”, instead of a power to change what we know about the world; an ascription we are already familiar with when speaking about technology and technological applications, but that is rather unusual for a characterisation of fundamental research—at least at a first glance.

## Techno/science idea(l)s and the discourse *about* science: Bacon, Bush and Bainbridge

Current representations of emerging research fields are characterised by striking allusions to power, control and intervention (for some examples, see Schmidt and Kastenhofer forthcoming). What do we make of such announcements after they have caught our eyes (and ears and imagination)? Clearly, they are difficult to ignore. Not only because of their powerful rhetoric, but also because these terms have been omnipresent in an ever growing flow of technoscience promotion and technoscience critique that propagates competing visions of future technoscience within tomorrow’s societies, of how we should see them and what they should look like. The rhetoric of the technosciences’ power to change, control and create our individual and societal future constitutes a narrative that is related to interventionist rather than representational science; it serves specific worldviews, political constellations and particular interests rather than others. It propagates the inescapable connection of technoscience and (interventionist) power, regardless of whether we have a pro or contra attitude towards it. In addition, if taken seriously, it asks for new ways of conducting, addressing and regulating emerging research fields. However, is it really a *new* narrative and is the act of launching a power rhetoric to propagate (a specific kind of) scientific research a *new* phenomenon? It seems so if compared to currently competing science narratives that painstakingly differentiate between science and technology as well as between basic and applied research and equate pure research with purely curiosity-driven quests for abstract knowledge about the laws of nature. It is also part of the new technoscience rhetoric itself to announce a fundamentally new science era.

With a wider historical scope, the association of science with power is not so new. The philosopher, scientist and statesman Francis Bacon stated already in 1620 that „Scientia et potentia humana in idem coincidunt” (Bacon 1620, Novum Organum Scientiarum, Book 1, Aphorism 3). He thereby established a programme of science as both, a quest for *true facts about* nature and a gain *of control of* nature; in line with current efforts to envision science and technology as two sides of the same (technoscience) coin. But when making sense of Bacon’s epistemological programme with reference to current contexts, one has to keep in mind that at this historical time, truth is still linked to science as well as religion (although this double reference starts to become problematic); gaining the right understanding of the world is both an individual duty as well as a societal aim; and power is gained by the exploration of new technical capabilities of control as well as by the exploration of new territories. Moreover, Bacon’s programme was formulated at a time when our current differentiation of science into arts, humanities, technical, formal, physical and life sciences and the respective disciplinary arrays did not exist. It can hence be read as a programme for all epistemic undertakings, highlighting the central epistemological role of empirical evidence and the dogmatic role of knowledge application. Bacon in his epistemology does not differentiate between the technical, the physical and the living world, but formulates one approach for all these contexts. Instead, an implicit differentiation into two general kinds of nature, natural laws and natural phenomena, seems to be highly relevant. Science gains its *potentia* via the understanding of natural laws; to understand laws equates to being able to successfully apply them in the manipulation of phenomena (Schmidt 2011). Phenomena on the other hand are not attributed any scientific relevance on their own; they are only important as stepping stones in the quest for acquiring knowledge about natural laws. Against this law/phenomena differentiation and prioritisation, modern physics, split into theoretical and experimental fields and closely related to the engineering sciences, can function as a role model for other research areas. Physics can serve as an example of a ‘hard science’, allowing for the formulation of abstract theories about natural laws that can be experimentally tested and hence put to force. Other, more idiographic areas such as natural history suffer from their ‘weak science’ character; if abstract knowledge is gained at all, it often does not lend itself to practical use because it takes the form of purely cognitive classifications of natural entities or describes historical processes that cannot be translated into experimental settings. Understanding, in Bacon’s view, has no merits on its own, it can even not occur on its own, without validation via successful application.

Vannevar Bush, more than 300 years later, again delineated a programme that was built upon the idea that science has the power to change the world (to the better). After World War II, in which technoscientific inventions and research programmes had already played a crucial role, Bush announced that science would continue to shape the international order in times of peace, leading to national health, prosperity and security:

Progress in the war against disease depends upon a flow of new scientific knowledge. New products, new industries, and more jobs require continuous additions to knowledge of the laws of nature, and the application of that knowledge to practical purposes. Similarly, our defence against aggression demands new knowledge so that we can develop new and improved weapons. This essential, new knowledge can be obtained only through basic scientific research. (Bush 1945)

Bush discerned the realm of basic research from the realm of applied research, the former being curiosity-driven and self-governed, the latter being application-driven and externally steered; he sees a linear, unidirectional link between both realms, leading from basic research via applied research to technological applications, industrial production and national wealth. Post-war science was embedded in a context where hard science equalled empirical research and science and religion no more competed for truth claims, science and industry had joined forces (also sharing major organisational characteristics in the regime of ‘Big Science’) in a quest for industrial applications and national competitiveness. This depiction of science, combined wit Bacon’s technoscientific ontology, seems to form the basis of every discourse on science and science policy until today (although it has also been contested as a heuristic model as well as a funding programme, cp. Stokes 1997).^12^ The trend to fund only (allegedly) powerful (or: problem-oriented, applicable, product-oriented, profitable, high-impact, translational, enabling and/or revolutionising) technoscience seems to unfold even further, both in the context of public as well as private funding. The UK government announced its plan to judge university research by impacts such as “the establishment of spin-out companies, influence on policy relating to the environment, or the development of products such as computer software or technology” (Gilbert 2010); the European Commission decided to “largely support research into climate change, food, security and health” with there “largest ever” budget for research of € 6.4-billion (Nature 2010), while the flow of industrial research funding (especially in biomedical fields) and the proliferation of public–private partnerships seem to continually increase.^13^ Hence, one could assume that the talk about powerful technoscience is taken seriously (to some extent) at the science policy level and results in specific decisions and adaptations.

The explicit funding aim to make an impact via research is also mirrored in statements coming from the scientific community, such as the announcement of a nanotechnology conference:

Nanotechnology—the ability to *understand and control matter with ultimate precision*—is the most *powerful and enabling* technology humankind has ever developed. Nanotechnology is used to create materials, devices and systems with fundamentally new properties and functions that will *change the world as we know it*. (Introduction to a Nanotech-Conference at Cornell University in June 2007,^14^ emphasis by the authors)

or a comment given by Craig Venter, an eminent scientist as well as promoter of synthetic biology, in an interview in May 2010, right after his announcement of having created the first artificial organism:

I think they [synthetic organisms] are going to potentially *create a new industrial revolution* if we can really *get cells to do the production we want*; if they could help wean us off of oil, and reverse some of the damage to the *environment* like capturing back carbon dioxide. We think some of the earliest applications people will see are new *vaccines*. We can make, in a day, new flu vaccines that have taken much longer to produce by conventional methods.^15^

But are such statements connected to real ambitions and changes within research practice? Or should they be understood as merely strategic statements that pave the way to get funding for business as usual research? One interesting interpretation of Bush’s programme, the post-war Big Science projects (such as the Human Genome Project) and recent NBIC initiatives (among the most prominent texts the report to the US National Science Foundation “Converging Technologies for Improving Human Performance” edited by Roco and Bainbridge 2002)^16^ is to see them as an attempt at legitimating Big Science in times of peace. The World War II Manhattan project that had resulted in the construction of the atom bomb served as a proof of principle that such new Big Science constellations opened up new dimensions of innovation and technoscientific potency. With the accomplishment of its technoscientific goal and the end of the war, new legitimisations for the allocation of huge amounts of resources into one joint technoscience project were required to prolong the era of Big Science that had just begun.^17^ These programmes were intertwined with a new way of science organisation. Moreover, this new kind of organising science relates to a new way of doing science. At the same time, the picture of science as a local, small size, socio-politically detached purely epistemic activity persisted, just as if speaking about (techno)science in a science policy context, (inter)national (techno)science initiatives and doing (techno)science in the laboratory were two completely different things.

## Techno/science idea(l)s in *doing* science: the contemplative, interventionist, constructionist and creationist stance

*Speaking about* science in the context of selling science and drafting science policies is not the same as *doing* science (although both activities are closely linked). Both, Bacon and Bush, are more famous for their ideas *about* science and their influence on science *policy* than for their own scientific research. When investigating the interventionist stance(s) of current (techno)scientific practices, standpoints and research approaches of active researchers—mirrored explicitly in statements or represented by specific research activities—are just as interesting. When scientists describe their professional work in interviews, on homepages or in research articles, the extent to which interventionist power is presented as constitutive for their work and which specific form this intervention takes vary profoundly. Scientists take on different positions towards intervention when making sense (to themselves or to others) of their research; these conceptions influence the way single actions are combined in order to achieve a related ultimate aim and they relate to bigger pictures of science, society and science governance such as those put forward by Bacon, Bush or Bainbridge. At least four crudely different ideal types that shape such accounts’ positioning towards intervention can easily be contrasted making use of empirical material gathered throughout research on epistemic cultures in previous and current research projects^18^: a contemplative, an interventionist, a constructionist and a creationist stance.

The ***contemplative stance*** aims at perceiving, apprehending and understanding.

The goal is to create insight [Erkenntnis] and to understand biological systems, no matter what I do. (Systems biologist, THCL, A I 4)

It makes use of field observation, collection, documentation, archiving and systematisation. Not to intervene and not to distort are instrumental for its success (cp. the complexity oriented culture in Kastenhofer 2007). It can be interpreted either as an the absence of targeted intervention and control or as a kind of power directed towards the self and one’s own mind and behaviour; disciplining, education and forming enlightened, modern citizens. It is present in writings of nineteenth century natural history, and can still be found in early ecology and related fields.^19^ Here, it is the quest for the understanding of a self-regulating and well regulated, balanced and harmonious natural world, “which harkened back to an earlier teleological view of nature as harmoniously regulated for the benefit of all in accordance with divine wisdom, with the new theoretical writing on evolution” (Kingsland 1991: 3). Forbes (1991/1887), a central figure in early American ecology, emphasises the ambition to paint a (representational) picture in an influential paper on “The Lake as a Microcosm” (ibid: 18):

And now if you will kindly let this suffice for the background or setting of the picture of lacustrine life which I have undertaken to give you, I will next endeavour-not to paint in the picture; for that I have not the artistic skill. I will confine myself to the humble and safer task of supplying you the pigments, leaving it to your own constructive imaginations to put them on the canvas.

He then goes on to describe the process of data collection, mimicking how any hiker might experience the ecological context:

When one sees acres of the shallower water black with water-fowl, and so clogged with weeds that a boat can scarcely be pushed through the mass; when, lifting a handful of the latter, he finds them covered with shells and alive with small crustaceans; and then, dragging a towing net for a few minutes, finds it lined with myriads of diatoms and other microscopic algae, and with multitudes of Entomostraca, he is likely to infer that these waters are everywhere swarming with life, from top to bottom and from shore to shore.

And concludes with the insight that “we have here an example of the triumphant beneficence of the laws of life applied to conditions seemingly the most unfavourable possible for any mutually helpful adjustment”.

Contemporary scientists still emphasise the establishment of “a feeling for the subject” or intuitive abilities based upon a non-invasive encounter between the researcher and the subject world:

We often go out relatively unencumbered and just look: What is actually happening outside? And then we allow ourselves to be surprised by what we find: We observe this and then try to evaluate our findings without looking for a specific systematic condition that has to be achieved. Thus, quite different objects are perceived at the same time, and we see how many unexpected developments there are. You come to realise how often what you observe differs from what you actually expected to find. It is our recurrent finding that self-organised natural systems are highly resistant to our planning. This aspect of self-organisation is perceived less as a disturbing factor that has to be eliminated, but rather as an actual characteristic of the systems. (Vegetation ecologist, NWK, I 4)

In this tradition, intervention can be interpreted as detrimental to the ability to gather an understanding (of life) which requires a holistic approach:

[The early holists] basically said, if you want to study a cell like I would do as a biochemist and molecular biologist, the moment I go into a living cell with a pipette, the cell dies. So you lose life. And they said, if you take the molecules out, you have lost the essence of life. (Systems biologist, THCL, UK I 1)

To put a more prosaic emphasis on understanding as the central research goal marks many of the contemporary accounts of technoscience and scientists. Understanding is then connected not as much to enlightenment as to curiosity-driven scientists and their playful and at the same time sincere interest in the laws of nature. Depending on the underlying further ambitions, understanding can then take on more of a contemplative flavour or more of an engineering flavour:

That means that the living nature of that organism, you cannot understand it independently of the other. So that’s basically life and the biology really emerges, arises when you go [to an organisational level] above the components and the components interact. (Systems biologist, THCL, UK I 1)

If we progress in our research, maybe we can better understand how leucocytes move; maybe one could then also understand, how this movement can be influenced with certain drugs and how we can then influence our immune defence. (Bio-mathematician, THCL, A I 7)

Just like intervening and understanding, predicting and understanding the behaviour of a living object can, but need not be mutually enhancing:

We actually have two aims: on the one hand we want to be able to *predict* the behaviour of systems; on the other hand we want to *understand* it. And there is always a slight discrepancy between these two aims. It is often easier to make predictions when one ignores the details. A spam filter, for example, searches for specific patterns, dollar symbols, specific words and the like. But it doesn’t know how Viagra works, or why people might buy Viagra or what it really stands for. We can also look for such patterns in biology and they allow us to make predictions. We can predict events without understanding them in detail. But we try to find a balance between prediction and understanding, so as to make useful predictions for instance about the most effective therapeutic strategy. (Bio-mathematician, THCL, UK I 9)

There are interesting shifts involved in the differentiation of varying modes of understanding: understanding is either based upon a specific disposition of the scientist (who strives to acquire a “feeling for the natural world”, to intuitively understand it), it is a situation the scientist is in (she/he is not fundamentally different but with or without understanding) or it is a property separable from the scientist, the object is either understood or not. Lyotard (1982) similarly speaks about an “intrinsic *exteriorisation* of knowledge in relation to the knower” (ibid: 16). “The old principle, according to which knowledge gain is inseparably linked to the education of the mind and the person itself, declines successively. Knowledge … ceases to be its own purpose; it loses its utility value”. (Lyotard 1979/2009: 31, transl. by the author). Moreover, subject and object switch from being intrinsically linked (understanding as an incorporated feeling for the object) to being fundamentally separated (understanding as *objectivation*) and to being causally linked (mechanistic understanding that allows for controlled intervention later onwards). And finally, understanding can point towards a holistic approach (understanding as antagonistic to intervention) or towards a mechanistic approach (understanding equals predictive and interventionist power and allows for a *mechanisation* of the object so that the object can be treated successfully as if it were a machine). These three processes, exteriorisation of knowledge, objectivation in the relation of subject and object, and mechanisation of the epistemic object, could be addressed as practices or stances in themselves; or at least, they are accomplished by specific actions taken by researchers. However, they are not treated in the same way as the contemplative or interventionist stance here, because they point towards more fundamental aspects of research and the shaping of research settings and can occur in practises related to understanding as well as intervention or construction. Exteriorisation of knowledge prepares the ground for its commodification and enabling character; objectivation prepares the ground for intervention from subject to object; mechanisation prepares the ground for mechanic intervention and construction.

In the above outline, the different modes of understanding share the joint trait that the scientist does not yet perform any action targeted at intervening in the object world other than for epistemic purposes. Nevertheless, various bigger pictures of the status of science in society have already been addressed: science as an education of the mind and personality or science as an enabler of instrumental interaction, intervention, control and prediction. The latter will now be discussed in further detail.

The ***interventionist stance*** of experimenting and controlling can be read as exerting a kind of sovereign power, reigning over the external, material world. Science intervenes in what is given by nature. Even if the natural sciences aim at understanding “Nature as it is”, the quest for understanding can involve an interventionist action, setting boundaries between the epistemic object and its environment, controlling certain parameters, manipulating the epistemic object to render it more amenable for experimentation or to observe the effects. The experimental objects, settings and contexts are staged, controlled, changed and manipulated. The ideas of control and manipulation are central to the interventionist approach and they are major aspects of the experimental sciences. Again, it has to be noted that in science experimental interventionism has traditionally been related to a quest for understanding, i.e. a quest that is just as close to a contemplative stance. It is even possible to depict this relation as a separation of work: the theoretical practices of a science take on a contemplative stance, the empirical practices take on an interventionist stance; or some aspects of research objects are controlled, others are left alone; or exploration equals a non-interventionist collection and processing of data, hypothesis testing is performed in highly controlled experiments.

More generally speaking, the turn towards experimental methods can be interpreted as a hinge between understanding-oriented and interventionist stances (cp. also Hoyningen-Huene 1989). It endows epistemic cultures with interventionist attitudes and capabilities, even if its immediate primary purpose is to deliver new insights. In line with this pattern, field sciences seem to harbour a less interventionist attitude than laboratory sciences and put less effort into control (cp. Kastenhofer 2007). The same is true for complexity oriented fields or fields that process a large amount of data in an exploratory way. Systems biologists, although they are not field scientists, give accounts that remind us of the ecologists’ account (given above):

There are the ones who work hypotheses-driven; they have an idea and do their research focussing exactly on this idea. And there are others who proceed in an undirected, unbiased, unprejudiced manner, so as not to exclude interpretations that maybe prove to be true later onwards. Of course, we always have hypotheses of the form that we say ‘if I treat the same plant in different ways, then I get a response, a difference at the molecular level’. This is assumed anyways. And then one could object that this is a totally undirected hypothesis. And this is partly the case. One always holds a certain idea as a researcher. But the beauty with this technique is that it does not allow for manipulation. You get the data, no matter if you like them or not. [Laughter] And now you have to cope with them. And you suddenly see, ‘there is an outcome, how should I explain it?’ It doesn’t fit to my hypothesis. Therefore, it is extremely important to be aware of that. The textbooks give us examples of cases where someone selected some aspects without looking more broadly at all related aspects. And this is correct on its own, self-contained; but, if you look further you suddenly see, ‘okay, but this is valid only under certain conditions, in other cases it is completely different’. (Systems biologist, THCL, A I 17)

**Fig. 1:**
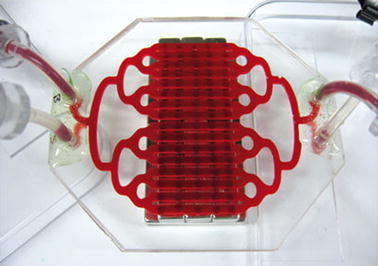
Between representation, intervention, control and construction: a ‘miniature version of the spleen’ on a chip ‘could speed tests of drugs and toxicity’ (Baker 2011: 661, picture reprinted by permission from the Wyss Institute for Biologically Inspired Engineering at Harvard University and Macmillan Publishers Ltd: Nature 461: 661, copyright 2011)

Experimental practices—although they aim at gaining new insights—not only promote interventionist attitudes and techniques, they also lead to the construction of new devices and epistemic objects (cp. Rheinberger 1997). Model organisms, test systems, experimental devices, all these include a constructionist aspect (for an example, see Fig. 1). In a Baconian mode, understanding, intervening and constructing are functionally connected.

I can see those fields, both normal biology and molecular biology, systems biology, as being completely important in trying to *understand* fundamentals of living systems. And as we increase the knowledge that we gain from those areas, it’s quite natural to then *flip onto the other side of the coin* and look to see if you can utilize that knowledge and information in a way to *build* new biological systems. (Synthetic biologist, THCL, UK I 7)

In a similar vein, the molecular biologist Francois Jacob (1988: 9) describes experimental systems more generally as “machines for making the future”. Still, to aim at “building new entities from scratch”, at building artificial objects or artefacts as an end in itself, represents a fundamental shift in a scientific culture. It equals a turn from a primarily epistemic to a first and foremost engineering culture.

The ***constructionist stance*** of designing and constructing refers to the engineering fields and the industrial power of material production. It considers the classic-modern engineering and science-based construction, not solely the intervention—the power to build artefacts and to construct material things. Whereas natural scientists consider the construction of artefacts mainly as a means to the end of a progress of representational knowledge, engineering scientists regard their ultimate goal as the construction of artefacts, things, machines, products and processes. Based on its major orientation towards the material production of artefacts, it can be discerned from another type of construction that entails further implications, namely the ***creationist stance*** of creating new forms of life within modern bio-technoscience. This position focuses on the creation of self-organising entities, emergent processes, living organisms or new species that (seem to) carry an intrinsic momentum of rest and dynamics in themselves, such as synthesised proteins, nano-bots or artificial life (cp. Schmidt 2008); in the socio-cultural context, they are endowed with an intrinsic value and cultural meaning. The creationist stance merges elements from the engineering sciences, the physical sciences and the life sciences.

If we somehow could bring the engineering discipline and how engineers approach problems, in design, in modelling and in how they build things into biological systems, that, to me, is synthetic biology. (…) If it’s true, the promise being that we can redesign nature to help us solve some of the big issues out there, it will have to be done through this kind of rigorous engineering approach. (…) That then takes you into how engineers do things and then you are into the engineering paradigm, you know, the parts, and the systems and the devices and the modules, and modelling, and computer simulation and testing and validation. (Synthetic biologist, THCL, UK I 7)

Following a constructionist paradigm, “Nature is neither tamed nor controlled, it is abolished” (Hohlfeld 1988: 66); following a creationist paradigm, the modern boundary between the natural and the artificial is obsolete. The creationist stance hence has two or three different roots and perspectives from which it can be defined: from the perspective of physics and classic-modern technology the radical innovation lies in the fact that the technical systems are attributed an intrinsic momentum of rest and dynamics, just like living systems; from the perspective of the life sciences, the ability to create “life from scratch” or to “construct new natural species”, hence the ability to produce living organisms and biological kinds just like physical artefacts and technical devices, entails another fundamental shift. It either results in a commodification of the living world (equating living and non-living entities) or in a radical empowerment of the involved technoscientists—up to the radical equation of technoscience and Creation, technoscientists and God. “Playing God” is maybe one of the most frequent accusations whenever technoscientists intervene in what is conceived as the core essence of life, be it genetic manipulation, creating new species, cloning or ending life. Thereby, on the subject as well as object level, meanings shift and categorical boundaries are blurred or at least questioned, as mirrored in media reports on Craig Venter’s project to “build a new life form from scratch”:

Venter was riding the waves again last week. He is close to making an artificial life form, very much an alpha male thing. It will, says Venter, conquer infection. Is he playing God? No, he’s more Adam, a new human beginning. He is, as he puts it, ‘the first chemical machine to gaze upon his own sequence’. He knows, in other words, his own DNA. He was the first man to decode the human genome, the announcement of which in 2000 was hyped as one of the great moments in history, like Galileo, Newton and Darwin rolled into one, but bigger. (Bryan Appleyard, The Sunday Times, October 28, 2007)^20^

Other debates focus on the question whether this stance can be realised at all on the practical level (even if it already exists as a paradigm and attitude).

Biologists who carry out experiments, would not be used to thinking in a kind of engineering way about something—the idea of building things from parts or genes and putting them together in different combinations and maybe testing each one on how they would work, that wouldn’t be a natural way of doing a biological experiment; even thinking about modelling a system in its own way so as to see if it will work efficiently … so biologists would have to come into that kind of feeling and biological engineering kind of thing and engineers of course will find it quite demanding because biological systems don’t behave in a predictable and robust way … The field in ten years time may go nowhere; might be like gene therapy; the bubble could burst and everyone may be saying, well what is the great deal about it? It could do, because it has been overhyped. (Synthetic biologist, THCL, UK I 7)

The reassessment of interventionist stances within emerging technosciences hence adds two further types to Ian Hacking’s representing and intervening: the constructionist and creationist approach. One could argue that the additional two types mirror a general shift or development of science that is related to the emergence of a technoscientific regime. The emergence of a technoscientific regime is pre-dated by the establishment of an interventionist experimental research culture, a culture that produced most of the preconditions of technoscientific practice without sharing their overall rationality. But whereas control and construction are instrumental within the experimental science, within technoscience the ultimate goal is a hybrid of understanding, intervening, constructing and creating; all four seem to be conflated into one meaning and practice: only what one can create, he or she can understand; only what is understood can be precisely controlled; only what one can control, he or she can really create (in an engineering manner). The central role of creationist power can be regarded as a major element, or at least a vision, of the technosciences. Creationist power emerges due to the success of technological reductionism and new directions in engineering sciences towards unification—facilitating a new openness towards natural sciences. In parallel, we also find the dissolution of boundaries by the natural science. Concepts of self-organisation, emergence and non-linearity, cultures of experimentation, dynamical system thinking and structure science (cp. Artmann 2010), stretch across many disciplines and open natural sciences to engineering approaches.

## Conclusion

This essay set out with the observation that besides the models of science as representing and intervening, as depicted by Hacking (1983), talk about the current technosciences is heavily characterised by allusions to power. This observation led to the formulation of three central questions: what exactly are the contemporary idea(l)s of (techno)science? How do these influence present day research cultures? What is their relevance in the socio-political context?

In a first step, we tried to identify concepts that might allow for analysing the relation between (explicit and/or implicit) (techno)science idea(l)s and (techno)scientific practice. This first step is seen as crucial because the conjunction between discourse *about* (techno)science, discourse and implicit presumptions *within* (techno)science and doing (techno)science is seldom addressed in current science studies whereas, more frequently, discursive phenomena are interpreted as primarily strategic acts in the sense that their performativity and reality is restricted to the arena of science policy. We identified Lyotard’s “Ideas” and Nordmann’s “organising myths” as possible proponents of conceptualising the interrelation of talking and doing technoscience, although both approaches have not yet been elaborated in much detail.

In a second step, we therefore looked for empirical cases in which the relation of science idea(l)s and scientific practice could be targeted. We chose three influential (techno)science (policy) programmes—the ones put forward by Francis Bacon in the seventeenth century, the one put forward by Vannevar Bush after World War II and the recent NBIC initiatives put forward by Mihail Roco, William Bainbridge and others. Additionally, we drew on depictions of scientific research stemming from interviews with contemporary scientists. We made use of these empirical examples to reconstruct the science idea(l)s immanent in these accounts. We found that both, Francis Bacon’s and Vannevar Bush’s models of (techno)science, include a representational and an interventionist momentum, but combine them in different ways. Moreover, the contemporary depictions of science helped to reconstruct two further stances besides a contemplative/representational and an interventionist stance: a constructionist and a creationist stance. The reconstruction of the different stances allows for discussing the link to specific research actions: it is argued that these stances do not preclude the singular actions taken (such as theorising, explaining, observing, controlling, representing, experimenting, modelling, designing, building, constructing), but shape how these actions are made sense of, referred to and combined within specific (techno-)scientific cultures.

The reconstruction of different interventionist stances has clearly been done in a very crude way in this paper. Still, we think it can serve as a starting point to address some interesting questions about the character and ramifications of technoscience, especially the relation of technoscience and power. It renders a more colourful picture than a twofold characterisation of (techno)science along the ideal types of representing and intervening would allow for [although admittedly Hacking’s (1983) analysis goes far beyond this binary characterisation] and further emphasises the plurality of pictures of (techno)science available today.

We tried to address the influence (techno)science idea(l)s exert on research practice. We also used the reconstruction of the four stances to differentiate how (techno)science policy actors and natural scientists frame the kind of power (techno)science exerts on the physical and social world. The four stances were formulated to depict different kinds of power. Interestingly, it depends on the overall socio-political regime how crucial these four kinds of power are seen.^21^ During the birth of modern science, the first stance might have been seen as elucidating the most radical change. To fundamentally change how the world is understood, to define man’s place in the world, to collectivise a specific understanding of the human, natural and physical condition via fundamental categorisations and to determine by which means understanding can be achieved and by which means the view of reality gets blurred and distorted were major sites of action in this contexts. What we called in a rather passive language ‘contemplative’ can hence also be framed as a powerful tool to (self-)discipline minds. The interventionist stance exerts power in a more visible and tangible way. The sources and targets of power are physical entities and they are usually distinct from each other, comparable to the distinct personae involved in the exertion of sovereign power. Constructionist power can be related to the industrial power of technological innovation, design and production. Creationist power is still of a different kind. The power to create new forms of life has so far been interpreted as an act and domain of God(s), a result of evolution (a process devoid of an actor) or mere fiction. Now that even scientists speak of their research as acts of creation (of new species, new life, etc.), marking the application of engineering practices to biological material and organisms, it is unclear what kind of socio-cultural meaning should be attributed to such an act. Is it to be understood as “playing God”? Or “tinkering with Nature” (a domain that should normally evolve without the involvement of actors)? And, moreover, what is the epistemic character of acts of creation? From an epistemological point of view, the American physicist Richard Feynman’s dictum “What I cannot build, I cannot understand” links the constructionist stance to an epistemic ambition. Francis Bacon’s dictum that scientific insight and human power (to control and create natural phenomena) are two sides of the same coin links the interventionist as well as the constructionist stance to an epistemic practice. But what is the link between creation and scientific understanding? Hohlfeld (1988: 64) links Bacon’s narrative to research that has been categorised as creationist in this paper: “Genetic engineers can hardly add something to Bacon’s ideal programme; the only difference is: they can now realise it”. (English translation by the authors) But one could also argue that Bacon’s programme does not differentiate epistemologically or ontologically between construction and creation or the physical and the living world; he rather differentiates between laws and phenomena.

Moreover, representational science has been criticised for misrepresenting reality, reifying reductionist interpretations or hindering pluralist perspectives. Interventionist science has been criticised for imperfect control, constructionist science for unforeseen side-effects of their constructs-in-context. But what about creationist science? If taken seriously, it could be criticised for “playing God” (although present day Western societies are based neither upon an explicit agreement that there is a god equipped with creationist power nor a collective definition of this power and its role in the world) or “messing with Nature” (a nature that is the legitimate or trusted site of creationist power). In more abstract terms, it is problematic because it points at lacunae in our current governance discourse (who is in charge of creation in secular knowledge societies?) and blurs the very boundaries our current governance regimes are based on [i.e. the boundaries between understanding, intervening and constructing, cp. Kastenhofer (2010)].

Mark Bedau, professor of philosophy and humanities and editor-in-chief of “Artificial Life”, touches upon this problem as discussant in “Creating the organisms that evolution forgot—An ‘Any Questions?’ debate on synthetic biology” organised at the London School of Economics on 26 November 2009.

[Accusations of ‘playing God’ are] going in one ear and out the other; that was the initial reaction of a lot of people, it was my initial reaction, but after thinking about this more, I’ve come to now adapt a different perspective (…). I think in fact that one of the reasons why people raise these worry is, it doesn’t actually come from religion and ethics, it comes with a concern about making things, changing our world in ways that have unpredictable and powerful consequences. I am not saying we shouldn’t do this, I am saying that we will feel comfortable about doing this only if you do it without hubris, only if you do it with a proper kind of humility and proper kinds of constraints and these constraints are roughly triangulated by a certain kind of picture of what a deity is.^22^

Alternatively, the new technosciences’ creationist character could be seen as mere hype and rhetoric. In our short account, we tried to argue that it would be too short-sighted to interpret technoscience programmes only as such, although the relations between *talking* technoscience and *doing* technoscience are complex and manifold. We see Lyotard’s concept of technoscience Ideas building upon Kant’s regulatory Ideas and Nordmann’s concept of “orientational myths” as useful staring points to conceptualise this relation, especially because they allow for the conclusion that technosciences never *fully* realise any of the four powers described above. Nevertheless, the relation between plural (techno)science idea(l)s as put forward by Hacking (1983), (techno)scientific practice and socio-political contexts deserves further and more systematic consideration if the relation between technoscience and power is to be understood in more depth, integrating epistemological, ontological and regulatory discourses.

As to this issue’s aim to probe the notion of technoscience, we conclude that the realisation of a creationist idea(l) within research practices might mark a new era. And, maybe more crucially, we can observe a shift in the context in which the four different stances are made sense of and further pursued. This shift is so fundamental, that it is difficult to delineate when looking at individual material practices and technoscientific output that builds upon earlier scientific achievements; but it becomes evident in the narratives that accompany science and technoscience, in current multidisciplinary research ensembles and in the ways (techno)science is made sense of in society.

## Footnotes

1 Although the latter term is plagued by an unconclusive discussion about its historical contextualisation as either a-modern, modern or post-modern, cp. also Forman (2007).

2 In the following text, “(techno)science” points at research that can be categorized either as science or as technoscience or both; science and technoscience will only be differentiated in the last chapter. The notation “idea(l)s” points at the fact that ideas about science are almost inseparable from ideas about sound science and ideal science.

3 The observation that the differentiation of science and technology, representing and interventing is simultaneously blurred and re-drawn resonates with Latour’s (1987) depiction of an “a-modern” dialectic between purification and hybridisation. But while Latour suggests an a-modern *de facto* dialectic combined with a modern idea(l) that emphasises purification, others suggest a post-modern idea(l) that emphasises hybridisation (cp. Lyotard 1979; Haraway 1997).

4 Barad (1998) uses the term epistem-ontology to do justice to the close link between scientific epistemologies (ideas about how a scientific understanding of the world is produced) and the ontologies (ideas about how the world is configured) held within scientific research.

5 The translator of the text, Bill Readings, notes at this point as an endnote: “I have preserved the term ‘Idea’ (capitalized), though Lyotard's Kantian account of the regulatory absolute (which exceeds the concept) has often been translated as ‘ideal.’ I avoid ‘ideal’ so as to forestall a too-ready accusation of ‘idealism.’” (ibid: 328).

6 See Böhme and Böhme (1983) for an analysis of the affinity between the Kantian and the Baconian ontology based on a presumably shared materialist perspective.

7 At the same time one could argue that, in the long run, they are themselves influenced by epistemic practices, social configurations and experiences that go with it. Herein lies a difference between Kant’s regulatory Ideas and contemporary co-constructivist conceptions of how our models of (ideal) science shape how we enact science.

8 As presented in Kuhn (1970). Both aspects are addressed in analyses of epistemic cultures (cp. Knorr-Cetina 1999).

9 This is especially worth emphasizing as talk of ‚myths’ in a pejorative way as a (often strategic) misrepresentation of reality also abounds at the very same time; cp. treatises about the “Myths of Agricultural Biotechnology“(Altieri, retrieved 10 May 2011), “The Frankenfood Myth” (Miller and Conko 2004), or about myths concerning “Public views on GMOs” (Marris 2001).

10 This is worth emphasizing to differentiate this standpoint from that of ontological (or radical) constructivism as well as from radical ontological understandings of what Baudrillard (1988) depicts as “hyperrealism” along which “Abstraction today is no longer that of the map, the double, the mirror or the concept. Simulation is no longer that of a territory, a referential being or a substance. It is the generation by models of a real without origin or reality: a hyperreal. The territory no longer precedes the map, nor survives it. Henceforth, it is the map that precedes the territory—precession of simulacra—it is the map that engenders the territory and if we were to revive the fable today, it would be the territory whose shreds are slowly rotting across the map.” (ibid: 166).

11 To (re-)link representing and intervening and to effectively and meaningfully manage this link has hence been part of the everyday practice and method of experimental research since its beginning [as mentioned in Hacking (1983)]; it is an integral part of the art of “making experiments work”.

12 It has also been contested (indirectly) in its positive attitude towards technology by influential fiction like Huxley’s *Brave New World* or Orwell’s *1984*, but without any ambition to vindicate its validity when it comes to the science-power-nexus.

13 The increase is not steady, stagnations or even decreases characterise the past few years; but between 1991 and 2010, an overall increase of the industry-financed share in relation to the government-financed share of the Gross Domestic Expenditure on R&D as a percentage of GDP becomes evident for the US, Europe as well as Japan (OECD 2010: 20).

14 http://www.cnf.cornell.edu/cnf_nanofutures.html, last accessed 10 May 2011.

15 http://www.bbc.co.uk/news/10132762, last accessed 28 December 2010, emphasis by the authors.

16 Furthermore, the National Nanotechnology Initiative or the European report “Converging Technologies—Shaping the Future of European Societies” (HLEG 2004) and various European national Nano-Initiative Action Plans.

17 The period of territorial politics (discovery and colonisation) and aggregation of national wealth (via biopolitics) during the Age of Enlightenment could be read as another, earlier Big Science era in which natural philosophers, conquerors, the mercantile marine and emperors joined forces, resulting (inter alia) in scientific discoveries and technological innovation that would not have been in reach without this specific socio-political constellation and channelling of resources.

18 Within the research projects “Nichtwissenskulturen” (abbr. NWK, 2004–2007, Univ. Augsburg) and “Towards a holistic conception of life? Epistemic presumptions and socio-cultural implications of systems biology” (abbr. THCL, 2010–2013, Institute of Technology Assessment at the Austrian Academy of Sciences, Research Centre for Biotechnology, Society and the Environment at the Univ. Hamburg) by one of the authors (K.K.).

19 Although early ecology already strived for experimental approaches and agricultural application and openly rejected the descriptive methods of natural history (Cittadino 1980).

20 http://www.timesonline.co.uk/tol/news/science/article2752196.ece, last accessed 10 May 2011.

21 Pestre (2003) in his historical analysis of the relation of science and society argues that scientific power has always been closely affiliated to political power. Only what power is and how it is exerted changes over time. If applied to the four ideal types reconstructed here, this would mean that all four types are connected to power.

22 Transcribed from recording available at http://www2.lse.ac.uk/publicEvents/events/2009/20090826t1656z001.aspx, last accessed 30 September 2011).
